# Controlling the propagation asymmetry of hyperbolic shear polaritons in beta-gallium oxide

**DOI:** 10.1038/s41467-023-40789-7

**Published:** 2023-08-28

**Authors:** Joseph Matson, Sören Wasserroth, Xiang Ni, Maximilian Obst, Katja Diaz-Granados, Giulia Carini, Enrico Maria Renzi, Emanuele Galiffi, Thomas G. Folland, Lukas M. Eng, J. Michael Klopf, Stefan Mastel, Sean Armster, Vincent Gambin, Martin Wolf, Susanne C. Kehr, Andrea Alù, Alexander Paarmann, Joshua D. Caldwell

**Affiliations:** 1 Vanderbilt University, Nashville, TN USA; 2 Fritz Haber Institute of the Max Planck Society, Berlin, Germany; 3 School of Physics, Central South University, Changsha, Hunan China; 4 Photonics Initiative, Advanced Science Research Center, City University of New York, New York, NY USA; 5 Institute of Applied Physics, TUD Dresden University of Technology, Dresden, Germany; 6 Physics Program, Graduate Center, City University of New York, New York, NY USA; 7 The University of Iowa, Iowa City, IA USA; 8 Helmholtz-Zentrum Dresden-Rossendorf, Dresden, Germany; 9 Attocube Systems AG, Munich, Germany; 10 NG NEXT, Northrop Grumman Corporation, Redondo Beach, CA USA

**Keywords:** Nanophotonics and plasmonics, Semiconductors

## Abstract

Structural anisotropy in crystals is crucial for controlling light propagation, particularly in the infrared spectral regime where optical frequencies overlap with crystalline lattice resonances, enabling light-matter coupled quasiparticles called phonon polaritons (PhPs). Exploring PhPs in anisotropic materials like hBN and MoO_3_ has led to advancements in light confinement and manipulation. In a recent study, PhPs in the monoclinic crystal β-Ga_2_O_3_ (bGO) were shown to exhibit strongly asymmetric propagation with a frequency dispersive optical axis. Here, using scanning near-field optical microscopy (s-SNOM), we directly image the symmetry-broken propagation of hyperbolic shear polaritons in bGO. Further, we demonstrate the control and enhancement of shear-induced propagation asymmetry by varying the incident laser orientation and polariton momentum using different sizes of nano-antennas. Finally, we observe significant rotation of the hyperbola axis by changing the frequency of incident light. Our findings lay the groundwork for the widespread utilization and implementation of polaritons in low-symmetry crystals.

## Abstract (web summary)

Hyperbolic phonon polaritons occurring in anisotropic materials exhibit strong light confinement and propagation directionality. Matson et al. report real-space imaging and control of recently discovered hyperbolic shear phonon-polaritons in beta-Ga2O3, arising from symmetry breaking in the dielectric response.

## Introduction

Crystalline structure largely defines the propagation of light in highly anisotropic materials, providing a means for controlling its behavior in conventional optical systems using polarizers, filters, and waveplates. In the infrared spectral range, where photon frequencies overlap with lattice resonances, crystalline structure strongly affects light propagation, with optical phonons dominating the dielectric response of a material. In polar materials, Coulomb interactions break the degeneracy between transverse (TO) and longitudinal optical (LO) phonons, while the strong net dipole moment of the TO phonon causes the material to also couple with resonant infrared light. This coupling is responsible for establishing the material-specific Reststrahlen band^1^ between the TO and LO phonon frequencies, within which light cannot propagate, and the material becomes highly reflective.

Under appropriate momentum-matching conditions, the optical phonons may couple to light to form PhPs that behave similarly to plasmon polaritons in metals^2,3^, albeit with much lower losses^3,4^ and within a narrower spectral region. However, the inherent coupling of PhPs to lattice vibrations also enables a far greater degree of nanophotonic control in materials with lower crystal symmetry. This was first demonstrated in quartz^5^ and hexagonal boron nitride^6,7^, where the significant structural anisotropy between in- and out-of-plane crystal axes results in energetically offset optical phonons. This anisotropy gives rise to different spectral bands supporting hyperbolic phonon polaritons (HPhPs). These HPhPs are not confined to the interface but instead propagate within the volume of the crystal at an angle defined by the open angle of the isofrequency hyperbola^8^, and support modes characterized by large momentum. Therefore, HPhPs offer stronger light confinement, as well as increased control over the propagation direction when compared to conventional surface polaritons. The increased control offered by HPhPs has enabled applications such as hyperlensing^8–11^, on-chip reconfigurable metasurfaces^12,13^, enhancing thermal emission^14^, and even mediating super-Coulombic dipole–dipole interactions^15^. In orthorhombic lattices with even lower crystal symmetry, all three lattice constants may differ (while remaining orthogonal). In these biaxial materials, such as MoO_3_, it has been shown that in-plane, in addition to out-of-plane hyperbolicity can be realized^16–18^, enabling further control over polariton propagation^19^, and even independent control over the wavevector and Poynting vector^20^.

Recently, we extended this concept to crystals with even lower symmetry, such as monoclinic β-Ga_2_O_3_ (bGO), which not only exhibits three axes with distinct lattice constants but also a non-orthogonality of the crystal basis in one lattice plane. The non-orthogonality of the axes within monoclinic crystals results in non-negligible off-diagonal elements in the dielectric permittivity tensor matrix within the monoclinic plane. As a result, in the presence of loss, the permittivity tensor can no longer be diagonalized via real rotations. However, the real part of the permittivity tensor can be diagonalized by a frequency-dependent rotation in the monoclinic plane^21–23^. This rotation of the permittivity tensor represents a frequency dispersion of the major polarizability axes (“axial dispersion”), while a purely imaginary off-diagonal permittivity component—the shear term—is retained. We explored the polariton behavior in bGO by leveraging theory and prism-coupled measurements in the azimuthal polariton dispersion in this material^22^. Both theory and experiment demonstrate strong axial dispersion, as well as shear, measured as an asymmetry of the polariton quality factor about the rotated optical axes due to the off-diagonal loss in the rotated frame. As a result of these fundamental differences from traditional hyperbolic polaritons, these modes were dubbed hyperbolic shear polaritons (HShPs).

The unique properties of HShPs are highly promising for a range of nanophotonic applications as they offer a greater degree of control over light propagation. On the one hand, slight changes in the incident frequency result in dispersion in both the HShP wavelength and propagation direction. On the other hand, the shear effect leads to a symmetry breaking of the near-field propagation, which was observed recently within CdWO_4_^24^. However, while this material is monoclinic, it exhibits a weak shear effect resulting from the small monoclinic angle (92.1°) that limits the ability to fully quantify this behavior. For bGO, this effect is significantly amplified as the monoclinic axis is offset at 103.7°, however, the phonon frequencies are all below ∼800 cm^−1^, which is beyond the accessible spectral range for direct imaging with traditional quantum cascade laser (QCL)-based scattering-type scanning near-field optical microscopes (s-SNOMs). Yet, if this challenge can be overcome with appropriate schemes, this symmetry breaking may be pushed to the extreme, where true unidirectional light propagation is achieved in a natural material.

In this work, we experimentally map the unique near-field propagation of HShPs in bGO in real space using far-infrared s-SNOM through integration with the external radiation from a free-electron laser (FEL). This latter component is critical as this allowed for direct imaging and quantification of the shear-induced asymmetry of the polariton propagation as a function of frequency over all Reststrahlen bands of bGO. The s-SNOM experiment allows us to directly image the asymmetry in the shear polariton propagation in real space. Specifically, this enables access to the large momentum components of the HShPs where large asymmetry was predicted^22^ but was inaccessible in prior prism-coupling studies due to the inherent limitations of the prism’s refractive index^25^. To launch the polaritons, here we use circular gold nano-antennas. We show experimentally and via simulations that we can distinguish propagation asymmetries arising due to the inherent shear effect from those induced by the oblique incidence illumination^26^. Furthermore, by applying nano-antennas of different sizes coupling to different momentum components of the HShPs, we demonstrate steeply increasing asymmetry with rising HShP momenta in agreement with theoretical predictions. Additionally, we observe significant rotations of the hyperbolic axis compared to the crystal axis as the excitation frequency is changed. Thus, we show that conveniently HShPs naturally provide the highest control over their propagation characteristics at the largest confinement conditions, ideally matching requirements for a range of nanophotonic applications.

## Results

**Fig. 1:**
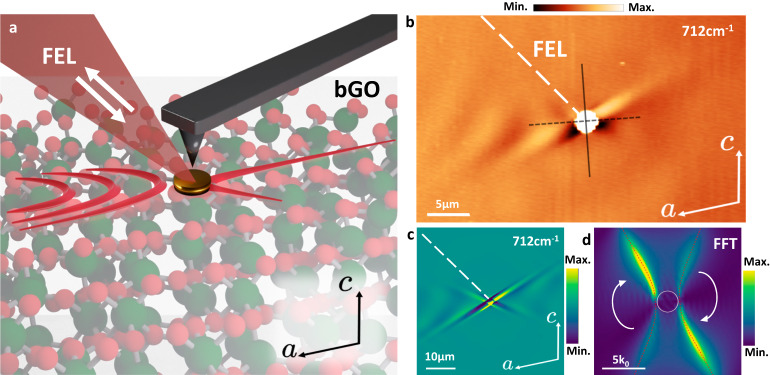
Real-space imaging of HShPs launching from infrared antenna on the surface of bGO observed via FEL-coupled s-SNOM. **a** Schematic of FEL-coupled near-field imaging experiment. **b** Experimental near-field image of HShPs launched by a 2 µm gold disc by the incident FEL beam (oriented along the dashed white line) at a frequency of 712 cm^−1^. **c** and **d** Finite element modeling of HShP launched by a 2 µm gold disc under similar launching conditions to the experiment shown in (**b**), in real-space (**c**) and Fourier space (**d**). Red dashed curves denote the dispersion of HShPs calculated from theory (Supplementary Note S1). The white arrows indicate the clockwise redistribution of intensity along each branch of the dispersion causing the asymmetry we observe in the momentum map, see Supplementary Fig. 3 for details.

To explore the shear propagation in real-space, we launch HShPs from gold disc antennas fabricated on the monoclinic surface of a bulk, [010]-face of a bGO crystal (Fig. 1a). These antennas allow us to launch HShPs along the surface of the bGO crystal, which we directly image using s-SNOM. Due to the low energy of optical phonons in bGO (primarily ranging from ∼200 to 750 cm^−1^), commercial infrared sources compatible with s-SNOM are limited or nonexistent in this range. Instead, we leverage a broadly tunable, narrow linewidth (FWHM ∼5 cm^−1^) free-electron laser (FEL). The FEL s-SNOM offers a frequency range spanning the infrared to THz (230–750 cm^−1^ in this work)^27–30^, allowing us to fully probe the spectral range of bGO HShPs.

Evidence of the axial dispersion and shear asymmetry of HShPs in real-space propagation is clear from Fig. 1b, which is characterized by strongly tilted wavefronts propagating at an angle skewed relative to the optical axis. In the experiment, the FEL is incident on the sample as shown by the white dashed line, at 45° off normal. To the left of the 2-µm diameter gold disc, we observe an in-plane hyperbolic, tilted wavefront propagating at an angle rotated away from the [100] axis. To the right, rather than fringes associated with hyperbolic polariton propagation, we observe ray-like modes propagating along one arm of the hyperbola (Fig. 1b). We note that while this ray-like propagation is similar to prior observations of so-called Ghost-polaritons in calcite^26^, the origins are from different physical phenomena. These results are in excellent agreement with our numerical simulations (Fig. 1c, d), as well as the theoretical HShP dispersion relation (red dashed curves in Fig. 1d). Fourier analysis of the disc-launched simulations (Fig. 1d) shows a clockwise redistribution of intensity to mostly one arm of the hyperbola due to the shear effect (by clockwise, we are referring to the azimuthal direction of intensity redistribution within each of the two branches of the hyperbolic dispersion in momentum space, see Fig. 1d and Supplementary Fig. 2 for an extended discussion).

Due to the oblique illumination in s-SNOM experiments, there is an inherent degree of orientation dependence in experiments. In uniaxial crystals like hBN, this results in a slight change in the polariton wavelength and real-space image depending on the orientation of the crystal edge with respect to the illumination^31^ as a result of the different phase-matching conditions between the polaritonic mode and the external excitation. Especially in crystals where the hyperbolic polariton axis is in-plane, the orientation dependence can be more important. In calcite, it was shown that the fringe vs. ray-like behavior of ghost polaritons depends on the orientation of illumination with respect to the hyperbolic axis^26^. As such, it is critical to determine that the asymmetric propagation observed in bGO is not merely a consequence of the oblique excitation, but instead a direct result of the anisotropic crystal structure.

**Fig. 2:**
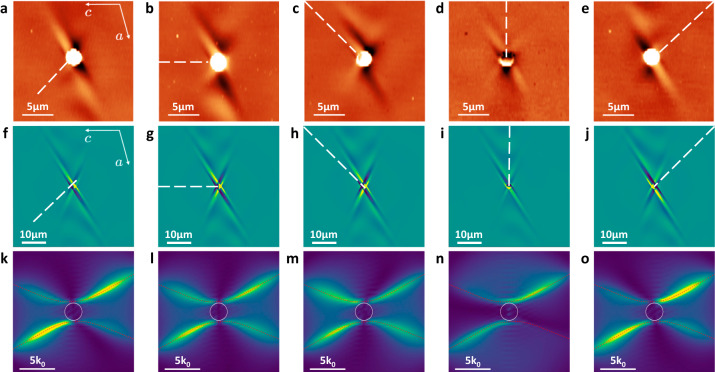
Dependence on illumination orientation of HShPs in a bulk bGO crystal. **a–e** Experimental near-field images of HShP propagation, launched by a 2 µm gold disc at a frequency of 712 cm^−1^, with FEL illumination rotated in 45-degree steps, as illustrated by a white dashed line. **f–j** Finite element modeling of HShP propagation from gold disc, with illumination conditions similar to (**a**–**e**). **k–o** Fourier transforms of **f**–**j**, illustrating the shear asymmetry in the polariton propagation. The red dashed curves in **k**–**o** are the dispersion of HShPs calculated from theory.

In bGO, the frequency dispersion of the polarizability axes makes aligning the illumination to the polariton axes impossible for more than a single frequency. As such, we explore the effects of illumination orientation on the HShP propagation by rotating the incident direction of the laser with respect to the crystal axes (Fig. 2a–e). In these s-SNOM plots, the FEL incidence direction clearly affects the visibility of polariton fringes, as well as the propagation length and intensity of the ray-like modes. We observe that in all cases the polariton propagation is clearly asymmetric. For incidence nearly along the hyperbolic wave asymptotes (Fig. 2a, c, e), we observe fringe-like propagation on one side, and ray-like behavior on the opposite. Additionally, after rotating by a full 180° (Fig. 2a, e) the shear tilt of the propagating wave is counter-clockwise in both orientations rather than mirrored across a symmetry axis—indicating the direction of shear is inherent to the crystal lattice and not to the incident direction. We note here that clockwise redistribution of intensity in momentum space, (Fig. 1d), results in counter-clockwise intensity redistribution in the real-space waveforms. When the incident beam is aligned with the *c*-axis, which nearly coincides with one polarizability axis at this frequency, and therefore is nearly perpendicular to the propagation direction (Fig. 2b), fringes are clearly visible to both sides of the antenna. In the case where the incident beam is nearly parallel to the propagation axis (Fig. 2d), no fringes are visible and instead ray-like modes with a clear, yet less pronounced asymmetry (in comparison to Fig. 2a, e) are launched weakly along the hyperbolic wave asymptotes.

The simulated field profiles with matching incidence orientations to the experiments (Fig. 2f–j) show varying degrees of asymmetry across the launcher. The incidence-dependent asymmetry is clear in the Fourier space plots (Fig. 2k–o). The asymmetry in the disc-launcher excitation originates from two mechanisms. The first contribution comes from the off-diagonal shear contribution to the permittivity tensor, causing the inherent asymmetry in polariton propagation, which can be best observed in near-field point-dipole excitations (Supplementary Fig. 7). The second mechanism is the orientation-dependent phase matching condition between the illuminated disc launcher and the excited polaritons. In fact, alignment of the incident light direction to the asymptotic lines of the hyperbolic waves where the shear effect intrinsically suppresses HShP propagation further enhances the propagation on the opposite side of the wave form (Fig. 2a, f, g). This corresponds to a strong enhancement of the clockwise intensity redistribution in the Fourier space. Therefore, in such a geometry we observe maximum asymmetry since shear and illumination effects add up constructively. On the contrary, excitation along the side of the waveform with intrinsically stronger intensity results in a nearly symmetric image, due to compensation of the asymmetries originating from the two mechanisms (see Supplementary Fig. 8, disc-launcher simulation with and without shear effect for more discussion). For excitation nearly along the polarizability axes (Fig. 2 b, g, l and d, i, n), the illumination-induced asymmetry is minimal and only the shear effect contributes to the asymmetry, leading to a slightly less prominent effect when compared to the cases in Fig. 2f, j. Yet despite the varying degrees of asymmetry observed here, HShP excitation is always biased in the same direction, demonstrating that the shear effect arising from the crystal anisotropy is dominant compared to any illumination effects that break the symmetry of the polariton propagation. Moreover, the dependence of the asymmetry upon the illumination orientation also highlights that the phase-matching mechanism is a crucial factor of control over the shear polariton propagation.

**Fig. 3:**
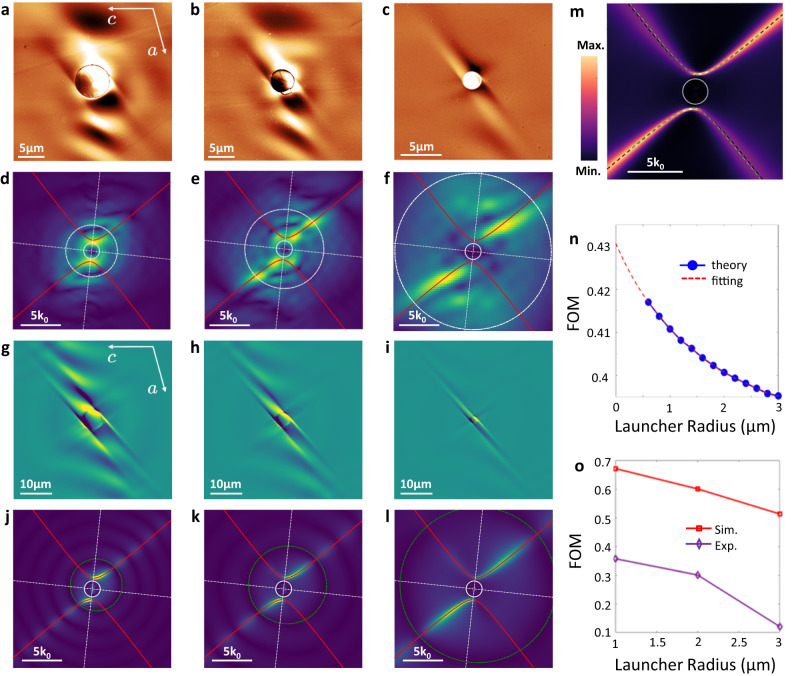
Momentum dependence of HShPs computed analytically and probed by illuminating gold resonators of different sizes. Experimental near-field images of HShP propagation, launched by **a** 6, **b** 4, and **c** 2 µm gold disc at a frequency of 720 cm^−1^, with illumination incident from the lower left corner. **d–f** Fourier transform of the experimental images in (**a**–**c**), with a zero-filling factor = 5. **g–i** Finite element modeling of HShP propagation from gold discs to match experiment (**a**–**c**). **j–l** Fourier transforms of (**g**–**i**). **m** Fourier transform of an electromagnetic simulation of an HShP excited by a local dipole illustrating the strong anisotropy at high excitation momentum. **n** Analytical FOM dependence on the launcher radius—extracted by integrating the damping rate along the path of HShPs dispersion which is cut off in momentum determined by the launcher radius. **o** Dependence of FOM on the launcher radius, extracted from Fourier transforms of the experimental and simulated field profiles (**a**–**c**, **g**–**i**). White circles in (**d**–**f**) and green circles in (**j**–**l**) intersecting dispersion of HShP determine the upper bound of the integration in the *k*-space of FOM calculation in Eq. (1) for experimental data and simulated data, respectively. The green circles in the simulated Fourier spectra are shifted by a small distance from the origin in k-space, due to the oblique illumination. In contrast, the white circles in the experimental Fourier spectra are symmetric with respect to the coordinate frame because we obtained these spectra from real-valued experimental data.

Our near-field experiments uniquely enable us to access polariton wavevectors that are much larger than the free space values. This is particularly interesting for HShPs, where theory predicts an increase of the shear effect with increasing momentum^22^. In our previous work, we were able to experimentally show such an increasing shear effect through prism-coupled studies. However, the overall asymmetry was small in those experiments due to the inherent limitations that restrict the technique to low wavevectors. Here, by using gold disc antennas with varying sizes ranging from 2 to 6 µm diameter (Fig. 3a–c), we can overcome such limitations and efficiently excite polaritons at much larger wavevectors to study the momentum dependence of shear in the strongly confined surface polariton regime.

Launched by a 6 µm diameter gold disc, HShPs propagate with tilted wavefronts from the bottom of the antenna (Fig. 3a), however, the ray-like mode is not clear. Around the disc, we observe a radical fringe pattern as well. As the disc size is decreased to 4 µm (Fig. 3b) and subsequently down to 2 µm (Fig. 3c), the radial fringes become less prominent, and the ray-like modes become more apparent. By taking a 2D Fast Fourier Transform (FFT) of the experimental images (Fig. 3d–f), we observe a clear progression towards a single branch of the hyperbolic dispersion—indicating that by launching at higher wavevectors, i.e. smaller disc size, the excited HShPs become not only increasingly directional and confined, but they are also effectively excited exclusively along one dispersion branch, further enhancing their directionality. Simulated field profiles and FFTs of the same-sized gold discs (Fig. 3g–l) match well with the experiment.

We can quantify the degree of shear in the polariton propagation based on complex-momentum eigenmode analysis to gain a more quantitative view of the momentum dependence of HShPs (Supplementary Note 1). To do this, we perform a line integral of the damping rate $\gamma=\frac{{q}_{{{{{\rm{{i}}}}}}}}{{q}_{{{{{\rm{{r}}}}}}}}$ along the arm of the HShP hyperbola in each quadrant of the *k*-space dispersion (Supplementary Fig. 1), and compare the integrals following two arms of the hyperbola to get a figure of merit for the shear asymmetry (FOM_Sh_):

1 $${{{{{{\rm{FOM}}}}}}}_{{{{{{\rm{Sh}}}}}}}\left({R}\right)=\frac{\left|{\sum }_{i}{\left(-1\right)}^{i}{\int }_{{k}_{{{{{\rm{{i}}}}}}}}^{{k}_{{{{{{\rm{R}}}}}}}}{{{{{{\rm{d}}}}}}k}\gamma \right|}{{\sum }_{i}{\int }_{{k}_{{{{{\rm{{i}}}}}}}}^{{k}_{{{{{{\rm{R}}}}}}}}{{{{{{\rm{d}}}}}}k}\gamma },$$

where ${k}_{{{{{\rm{{R}}}}}}}$ is the cut-off radius in momentum space determined by the radius of the disc launcher, and ${k}_{{{{{\rm{{i}}}}}}}$ is the intersection of the rotated hyperbola axis and hyperbolic dispersion in the according quadrant. Equation (1) allows us to calculate the FOM_Sh_ from the analytical description of HShPs by analyzing propagation losses of the shear polaritons and considering the wavevector shift due to the illumination angle of incidence (see Supplementary Note 2 for details of the FOM analysis). We see that the FOM_Sh_ obtained using Eq. (1) on the analytically determined $\gamma$ is inversely proportional to the launcher size, suggesting an increase in the asymmetry with increasing wavevector magnitude (Fig. 3n). Moreover, we are also able to extract FOM_Sh_ from our direct measurement and simulation of the HShPs launched from the different sized launchers by integrating over the intensities along the dispersion in the experimental and simulated FFT maps. We observe a similar relationship to the analytical case, but the change in the FOM_Sh_ with launcher size appears to be significantly larger in amplitude (Fig. 3o). This is consistent with the analysis associated with Fig. 2: two mechanisms contribute to the measured asymmetry upon launcher excitation, while the theoretical calculations only account for the shear effect of the HShPs, thus FOM_Sh_ from the simulations are expected to be larger than the ones predicted from theory. We observe a change in the magnitude of FOM_Sh_ between experiment and simulation, which may result from the lack of the s-SNOM tip in simulations, yet we still observe a strong launcher-size dependent FOM_Sh_ experimentally. From these results, we can quantitatively show that shear increases significantly with reduced disc size and, thus, under conditions where larger polariton momentum components are excited, providing a promising avenue for full symmetry control of strongly confined HShPs.

**Fig. 4:**
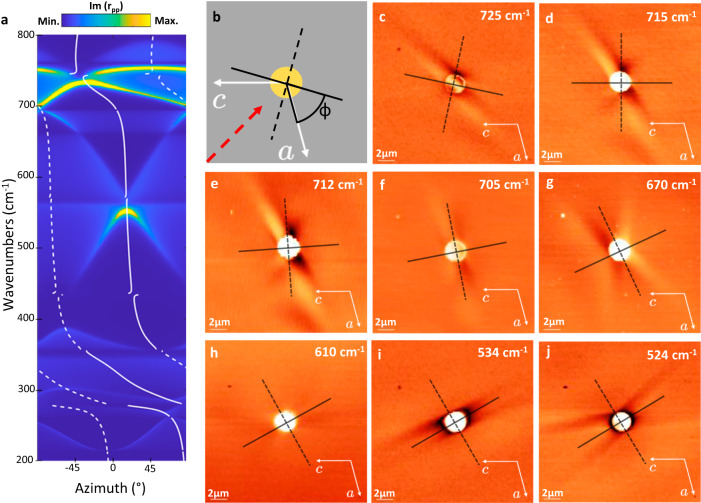
Frequency-dependent rotation of HShP propagation. **a** Plot of the permittivity rotation angle ф (white lines), overlaid on a transfer matrix method (TMM) calculation of the azimuthal polariton dispersion (via the imaginary part of the reflection coefficient *r*_pp_) for 2 µm gold discs. **b** Schematic of the frequency-dependent rotation of the polariton axis. **c–j** Rotated polariton axis plotted, as in **b**, against s-SNOM collected near-field plots of HShPs launched by a 2-µm diameter gold disc, at varying frequencies (Scale bar = 2 µm).

Aside from the shear asymmetry that we have explored above, HShPs are also defined by a strong rotation of the hyperbola axis with changing frequency that we refer to as axial dispersion^22^. To quantify this, we calculate the polarizability axes (white solid and dashed lines), which are rotated by an angle ф^21–23^ (Fig. 4a), and overlay the dispersive axes over a TMM calculation of the polariton dispersion in semi-infinite bGO (as we calculated previously^22^, but with the momentum calculated for a 2-µm disc launcher rather than a prism). We acquired experimental real-space polariton propagation over a wide range of incident frequencies (Fig. 4c–j). The polaritons propagate in asymmetric hyperbolic patterns, aligned around the frequency-dependent rotating polariton axes (shown in black), while the propagating fringes (when visible) are tilted due to the shear. At different frequencies, we observe varying degrees of ray-like propagation vs tilted polariton fringes. This is due in part to the inherent dispersion of the polariton behavior and specifically to the frequency-dependent magnitude of the shear but is also affected by the interplay between axial dispersion and the fixed illumination orientation as discussed in Fig. 2. To verify that the excited polaritons are HShPs at various frequencies, we provide disc-launcher simulations for select frequencies in Supplementary Fig. 10 and confirm their shear hyperbolic nature. Our data clearly demonstrates that bGO supports sub-diffractional (∼*λ*_0_/4, Supplementary Fig. 3), strongly asymmetric directional polaritons with long propagation lengths (>10 µm, Supplementary Fig. 3) across a wide spectral range in the far-infrared.

In conclusion, we have explored symmetry breaking at the nanoscale in the near-field propagation of hyperbolic shear polaritons in bGO. These results demonstrate near-field measurements of the nano-scale real-space HShP propagation in bGO, exhibiting a strong rotational asymmetry and a wide tunability of the propagation direction as a result of the monoclinic structure of the underlying crystal lattice and strong anisotropy in the phonon resonances, leading to giant nano- to microscopic shear effects. We have demonstrated that polaritons in monoclinic crystals offer significant control over polariton propagation directionality via excitation frequency, illumination direction, and disc launcher size, exceeding that offered by higher symmetry polaritonic systems. Furthermore, we theoretically and experimentally reveal a hidden synergy between shear, and hence polaritonic directionality, with increasing HShP confinement. We believe that the exquisite control over the degree of symmetry-breaking offered by HShPs via shear and axial dispersion provides an avenue for enhanced efficiency of light-guiding at deeply sub-wavelength scales.

## Methods

### Sample preparation

We used commercially available wafer samples of [010] β-Ga_2_O_3_, doped with Fe in order to compensate for inherent free carriers (∼10^12^ cm^−3^). Gold antennas were fabricated on the surface of the wafer via standard electron beam lithography. The patterns were written into bilayer EBL resist (PMMA 495/A3 ∼50 nm, PMMA 950/A3 ∼100 nm), which was spin-coated onto the substrate, and then developed in MIBK. A metal film (5 nm Ti, 50 nm Au) was deposited by e-beam evaporation, followed by a standard liftoff procedure.

### FEL s-SNOM

For the near-field imaging of the polaritons, we used a commercial s-SNOM system from Neaspec, coupled to the free-electron laser FELBE at the Helmholtz–Zentrum Dresden–Rossendorf. In contrast to typical s-SNOM experiments with highly stable commercial laser sources, we rely on self-homodyne measurements rather than pseudo-heterodyne due to the relative instability of the FEL. This results in intermixed amplitude and phase channels. To account for this, we consider the detected optical intensity:

2 $$I={\left({\vec{E}}_{{{{{{\rm{nearfield}}}}}}}+{\vec{E}}_{{{{{{\rm{farfield}}}}}}}\right)}^{2}={{E}_{{{{{{\rm{nearfield}}}}}}}}^{2}+{{E}_{{{{{{\rm{farfield}}}}}}}}^{2} \\+2{E}_{{{{{{\rm{nearfield}}}}}}}{E}_{{{{{{\rm{farfield}}}}}}}\, {{\cos }}\left({\varphi }_{{{{{{\rm{nearfield}}}}}}}-{\varphi }_{{{{{{\rm{farfield}}}}}}}\right)$$

Here, the first term (${{E}_{{{{{{\rm{nearfield}}}}}}}}^{2}$) is typically too small to be significant, the second term $({{E}_{{{{{{\rm{farfield}}}}}}}}^{2})$ is not affected by the tip oscillation and is therefore filtered by the lock-in amplifier, leaving the third term as our measurement signal. For simplicity, we can set ${\varphi }_{{{{{{\rm{farfield}}}}}}}=0$ for one wavelength. Then our recorded signal is approximately:

$${S}_{{{\det }}}=2{E}_{{{{{{\rm{nearfield}}}}}}}{E}_{{{{{{\rm{farfield}}}}}}}\, {{\cos }}\left({\varphi }_{{{{{{\rm{nearfield}}}}}}}\right)$$

Thus, our signal in the self-homodyne scheme is a mixture of the near-field and far-field response with the phase. Figures 1, 2, and 4 show demodulations of *S*_det_ at the second harmonic of the tip oscillation frequency and are analyzed as discussed above.

### OPO s-SNOM

In Fig. 3, we leverage a commercial s-SNOM system from NeaSpec, coupled with a widely tunable mid-IR OPO laser source covering a spectral range from ca. 1.4 to 18.2 µm wavelength (wOPO, www.attocube.com/neaspec).

### COMSOL simulations

COMSOL version 6.0 was used for simulating both point dipole excitation and disc launcher excitation of shear polaritons at the interface of [010] β-Ga_2_O_3_ and air. Scattering boundary conditions were used on the boundaries to absorb all outgoing radiation. The area of the interface between air and the bGO slab was (80$\times$80) μm, such that shear polaritons are sufficiently damped when it reaches the boundary so as not to influence the results.

For near-field simulation, a point dipole source was placed 200 nm above the surface of the material. For disc-launcher simulations, a plane wave source with p-polarization and a fixed incident angle of ${45}^{\circ }$ is placed 6 μm above the interface. The gold disc launcher is 2 µm in diameter and 100 nm in thickness. In far-field simulations, to filter out the background due to plane wave illumination and reflection from the interface and extract the near field distribution of polaritons from disc launcher excitation, we performed twice the same simulation with and without the presence of the disc launcher, respectively, and calculate the difference of fields in the two cases evaluated at 100 nm above the interface.

### Supplementary information

Supplementary Information

## Data Availability

Experimental and simulation data for this study can be accessed via Zenodo: 10.5281/zenodo.8162810.

## Code availability

The codes that support the findings of this study are available from the corresponding authors upon reasonable request.
