# Supplementary material for: Controlling the propagation asymmetry of hyperbolic shear polaritons in beta-gallium oxide
Source: Nat Commun. 2023 Aug 28;14:5240. doi: 10.1038/s41467-023-40789-7 (PMC10462611; doi:10.1038/s41467-023-40789-7)
Supplement: Supplementary file 1 — Supplementary Information [file 41467_2023_40789_MOESM1_ESM.pdf]

Supplementary Information:

Controlling the Propagation Asymmetry of Hyperbolic Shear Polaritons in Beta-Gallium Oxide

Joseph Matson*, Sören Wasserroth*, Xiang Ni*, Maximilian Obst, Katja Diaz-Granados, Giulia Carini, Enrico Maria Renzi, Emanuele Galiffi, Thomas G. Folland, Lukas M. Eng, J. Michael Klopf, Stefan Mastel, Sean Armster, Vincent Gambin, Martin Wolf, Susanne C. Kehr, Andrea Alù, Alexander Paarmann, Joshua D. Caldwell

Supplementary Note 1: Complex-momentum eigenvalue analysis for surface shear polaritons

We assume plane wave solution $e^{i(k_x x+k_y y-i\kappa_z-\omega t)}$ for the components of hyperbolic shear polaritons (HShPs) in bGO which exponentially decay away from the interface, thus source-free Maxwell equations read

$$\begin{pmatrix} \epsilon_{xx}-(-\kappa_z^2+k_y^2) & \epsilon_{xy}+k_xk_y & -ik_x\kappa_z \\ \epsilon_{xy}+k_xk_y & \epsilon_{yy}-(-\kappa_z^2+k_x^2) & -ik_y\kappa_z \\ -ik_x\kappa_z & -ik_y\kappa_z & \epsilon_{zz}-(k_y^2+k_x^2) \end{pmatrix}\begin{pmatrix} E_x \\ E_y \\ E_z \end{pmatrix}=0, \quad (1)$$

We then have the polynomial equation by solving Eq. (1)

$$\epsilon_{zz}\kappa_z^4+\left[\epsilon_{zz}\left(\epsilon_{xx}+\epsilon_{yy}\right)-\epsilon_{zz}k_x^2-\epsilon_{zz}k_y^2-\left(\epsilon_{xx}k_x^2+\epsilon_{yy}k_y^2+2\epsilon_{xy}k_xk_y\right)\right]\kappa_z^2 \\ +\left(\epsilon_{zz}-k_x^2-k_y^2\right)\left[\epsilon_{xx}\epsilon_{yy}-\epsilon_{xy}^2-\left(\epsilon_{xx}k_x^2+\epsilon_{yy}k_y^2+2\epsilon_{xy}k_xk_y\right)\right]=0. \quad (2)$$

Therefore, the solutions of κ_z are obtained as

$$\kappa_\pm^2=\frac{-b\pm\sqrt{b^2-4ac}}{2a}, \quad (3)$$

where

$$a=\epsilon_{zz}, \\ b=\epsilon_{zz}\left(\epsilon_{xx}+\epsilon_{yy}\right)-\epsilon_{zz}k_x^2-\epsilon_{zz}k_y^2-\left(\epsilon_{xx}k_x^2+\epsilon_{yy}k_y^2+2\epsilon_{xy}k_xk_y\right), \\ c=\left(\epsilon_{zz}-k_x^2-k_y^2\right)\left[\epsilon_{xx}\epsilon_{yy}-\epsilon_{xy}^2-\left(\epsilon_{xx}k_x^2+\epsilon_{yy}k_y^2+2\epsilon_{xy}k_xk_y\right)\right]. \quad (4)$$

To guarantee the physical solutions, the solutions in Eq. (3) need to satisfy $b^2-4ac\geq 0$, and $\kappa_\pm^2\geq 0$ so two unphysical solutions are eliminated. To simplify the expression of eigenfield solutions, we apply rotation to Maxwell equations in the xy plane: $\mathrm{R}(\phi)=\begin{pmatrix} \cos(\phi) & \sin(\phi) & 0 \\ -\sin(\phi) & \cos(\phi) & 0 \\ 0 & 0 & 1 \end{pmatrix}$, and also replace k_x and

k_y with the following relations $k_x = qcos(\phi), k_y = -qsin(\phi)$, thus the momentum vectors of surface polariton in the rotated basis become $\boldsymbol{k}_\pm = (q, 0, -i\kappa_\pm)$. Permittivity components after rotation turn into

$$\epsilon_{xx}^t = \frac{1}{2}\left(\epsilon_{xx} + \epsilon_{yy} + \left(\epsilon_{xx} - \epsilon_{yy}\right)\cos(2\phi) + 2\epsilon_{xy}\sin(2\phi)\right),$$

$$\epsilon_{yy}^t = \frac{1}{2}\left(\epsilon_{xx} + \epsilon_{yy} + \left(-\epsilon_{xx} + \epsilon_{yy}\right)\cos(2\phi) - 2\epsilon_{xy}\sin(2\phi)\right),$$

$$\epsilon_{xy}^t = \epsilon_{xy}\cos(2\phi) + \left(-\epsilon_{xx} + \epsilon_{yy}\right)\cos(\phi)\sin(\phi).$$

$$\epsilon_{zz}^t = \epsilon_{zz}. \tag{5}$$

Under the rotated basis, Maxwell equations are simplified as

$$\widetilde{\mathrm{M}}_\pm = \begin{pmatrix} \epsilon_{xx}^t + \kappa_\pm^2 & \epsilon_{xy}^t & -i\kappa_\pm q \\ \epsilon_{xy}^t & \epsilon_{yy}^t - (-\kappa_\pm^2 + q^2\,) & 0 \\ -i\kappa_\pm q & 0 & \epsilon_{zz}^t - q^2 \end{pmatrix} \begin{pmatrix} E_x^t \\ E_y^t \\ E_z^t \end{pmatrix} = 0, \tag{6}$$

As a result, the general forms of eigenmode in bGO can be expressed as

$$\boldsymbol{E}_+^{\boldsymbol{t}} = [m_{22}^+ m_{33}^+, -m_{21}^+ m_{33}^+, -m_{22}^+ m_{31}^+],$$

$$\boldsymbol{H}_+^{\boldsymbol{t}} = \left[-i\kappa_+ e_y^+, i\kappa_+ e_x^+ + q e_z^+, -q e_y^+\right]$$

$$\boldsymbol{E}_-^{\boldsymbol{t}} = [m_{22}^- m_{33}^-, -m_{21}^- m_{33}^-, -m_{22}^- m_{31}^-]$$

$$\boldsymbol{H}_-^{\boldsymbol{t}} = \left[-i\kappa_- e_y^-, i\kappa_- e_x^- + q e_z^-, -q e_y^-\right], \tag{7}$$

Similarly, the solutions of HShPs in the air have the plane wave form $e^{i(k_x x + k_y y + i\kappa_{air} - \omega t)}$, where $\kappa_{air} = \sqrt{q^2 - \epsilon_{air}}$. The eigenmodes can be decomposed into two independent solutions

$$\text{TM: } \boldsymbol{E} = [-i\kappa_{air}, 0, q], \boldsymbol{H} = [0, \epsilon_{air}, 0];$$

$$\text{TE: } \boldsymbol{E} = [0,1,0], \boldsymbol{H} = [i\kappa_{air}, 0, -q]. \tag{8}$$

To procure the isofrequency contours (IFCs) of HShPs, we impose the continuous conditions for tangential fields at the boundary $z = 0$ and obtain the linear equations

$$\widehat{D}\begin{pmatrix} a_+ \\ a_- \\ a_{TM} \\ a_{TE} \end{pmatrix} = 0, \tag{9}$$

Where the matrix $\widehat{D}$ is

$$\hat{D} = \begin{pmatrix} e_x^+ & e_x^- & -i\kappa_{air} & 0 \\ e_y^+ & e_y^- & 0 & 1 \\ h_x^+ & h_x^- & 0 & i\kappa_{air} \\ h_y^+ & h_y^- & \epsilon_{air} & 0 \end{pmatrix}, \tag{10}$$

and $a_{\pm}$ and $a_{TM/TE}$ are the coefficients of wave components in bGO and air, respectively. For the lossless case, IFC dispersion of the surface polariton in k-space is obtained from the secular equation $\det[D] = 0$. Since we aim to study the eigenmodes and obtain IFC of shear polaritons supported by the lossy and anisotropic bGO, we have to adopt the complex-valued form for their in-plane momentum vector $\boldsymbol{q}_{\mathrm{c}} = \boldsymbol{q}_{\mathrm{r}} + i\boldsymbol{q}_{\mathrm{i}} = (k_x + i\kappa_x, k_y + i\kappa_y)$, where $\boldsymbol{q}_{\mathrm{r}}$ and $\boldsymbol{q}_{\mathrm{i}}$ are not necessarily in parallel. Particularly, we impose the condition that $\boldsymbol{q}_{\mathrm{i}}$ follows the same direction as the one of in-plane Poynting vector: $\boldsymbol{q}_{\mathrm{i}}||\boldsymbol{S}_{||}$ (The detailed study of this condition will be present in somewhere else). Consequently, we can solve the complex-valued dispersion of shear polaritons with enough number of equations

$$f_1(k_x, \kappa_x, k_y, \kappa_y) = \Re(\det[D]) = 0;$$
$$f_2(k_x, \kappa_x, k_y, \kappa_y) = \Im(\det[D]) = 0. \tag{11}$$

The damping rate $\gamma = |\boldsymbol{q}_{\mathrm{i}}|/|\boldsymbol{q}_{\mathrm{r}}|$ of shear polaritons can be evaluated from the complex-valued solutions in Eq. (11).

Supplementary Note 2: FOM calculation explanation

To quantify the shear effect of the hyperbolic polaritons both theoretically and experimentally, we propose a global figure of merit ($\mathrm{FOM_{Sh}}$) for the shear asymmetry as a function of disc radius which reads

$$\mathrm{FOM_{Sh}}(R) = \frac{\left|\sum_i (-1)^i \int_{k_i}^{k_R} dk\gamma\right|}{\sum_i \int_{k_i}^{k_R} dk\gamma}, \tag{12}$$

Where the line integration follows the path of IFCs of HShPs calculated from Eq. (11). The upper bound in the integration is

$$k_R/k_0 = \frac{\lambda}{\sqrt{2}R}, \tag{13}$$

which is the radius of white circle in k-space in Supplemental Figure 1, λ is the free space wavelength, R is the radius of the disc launcher. The lower bound in the integration k_i is the intersection of the rotated hyperbola axis and hyperbolic dispersion in the according quadrant, as shown in Supplemental Figure 1. We are also able to extract this FOM_{Sh} from our direct measurement and simulation of the HShPs launched from the different sized launchers (detailed in Supplementary Figure 8-9). However, we need to account

for the oblique incidence shifting the dispersion in k-space. We account for this by shifting k_R by the amount:

$$\Delta\boldsymbol{k} = k_0 \cos(\theta_{\text{inc}})\big(\cos(\phi), \sin(\phi)\big) \tag{14}$$

Where ϕ is the azimuthal angle of the beam incidence, and θ_{inc} is the angle of the incident beam from the sample surface. This shifted momentum is indicated by the green circles in Figure S1.

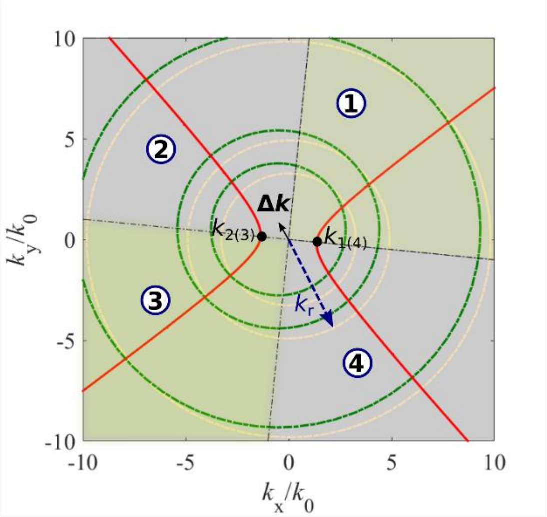

Supplementary Figure 1 – Rotated k-Space Quadrants for FOM calculation. *The schematic of quadrant division in k-space, and the cut-off radii (white circles or green circles with shift $\Delta\boldsymbol{k}$) correspond to excitation limitation of various discs launchers in real space. The red curves are IFCs of HShPs produced from the complex-momentum eigenmode study in Supplementary Note 1.*

Supplementary Note 3: Description of shear "clockwise" vs "counter-clockwise" bias

In the manuscript, we refer to the orientation of the shear effect by the counter-clockwise redistribution of the polariton intensity within the polariton hyperbola (Supplementary Fig. 2b). This counter-clockwise orientation also describes the tilt of the polariton wavefront (Supplementary Fig. 2a).

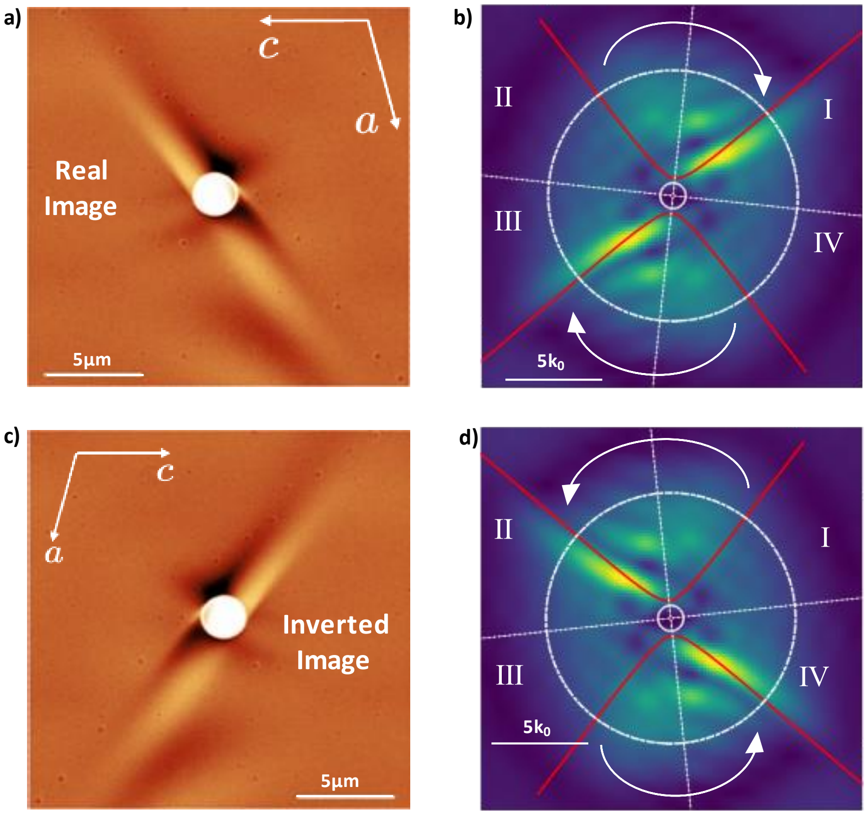

Supplementary Figure 2 – Orientation of Shear. *a) Experimental image from main figure 3c – launched at 720cm^{-1} by a 2 μm gold disc. b) Fourier transform of a (from Main figure 3f), with white arrow indicating the shear bias of the polariton. This corresponds to a left-handed shear of the polariton dispersion about the [010] axis. c-d) Fake images, from flipping (a-b) about the vertical axis. This illustrates the "counter-clockwise" bias we would observe on a [0-10] surface, which would still be a left-handed rotation about [010].*

Supplementary Note 4: Analysis of propagation lengths and polariton wavelength

In this section, we briefly analyze the polariton wavelengths and propagation lengths. In the FEL s-SNOM, we took large area measurements of the polaritons launched by a 2μm disc so that the polariton fully decays in the scan area (Supplementary Fig. 3 a,b). By nature of in-plane hyperbolic polaritons propagating from a disc antenna, there is not a clear point from which to extract the polariton features, however we can get a rough view of the polariton characteristics by taking linecuts of the field profile at different angles from the antenna.

Here, we have taken three linecuts distributed through the hyperbolic propagation and fit to an exponentially decaying sine wave.

$$y(x) = y_0 + Ae^{-\frac{x}{t_0}} \sin\left(\pi \frac{x - x_c}{w}\right) \tag{15}$$

Which gives us w for the polariton wavelength, and t_0 for the decay length of the polariton propagation.

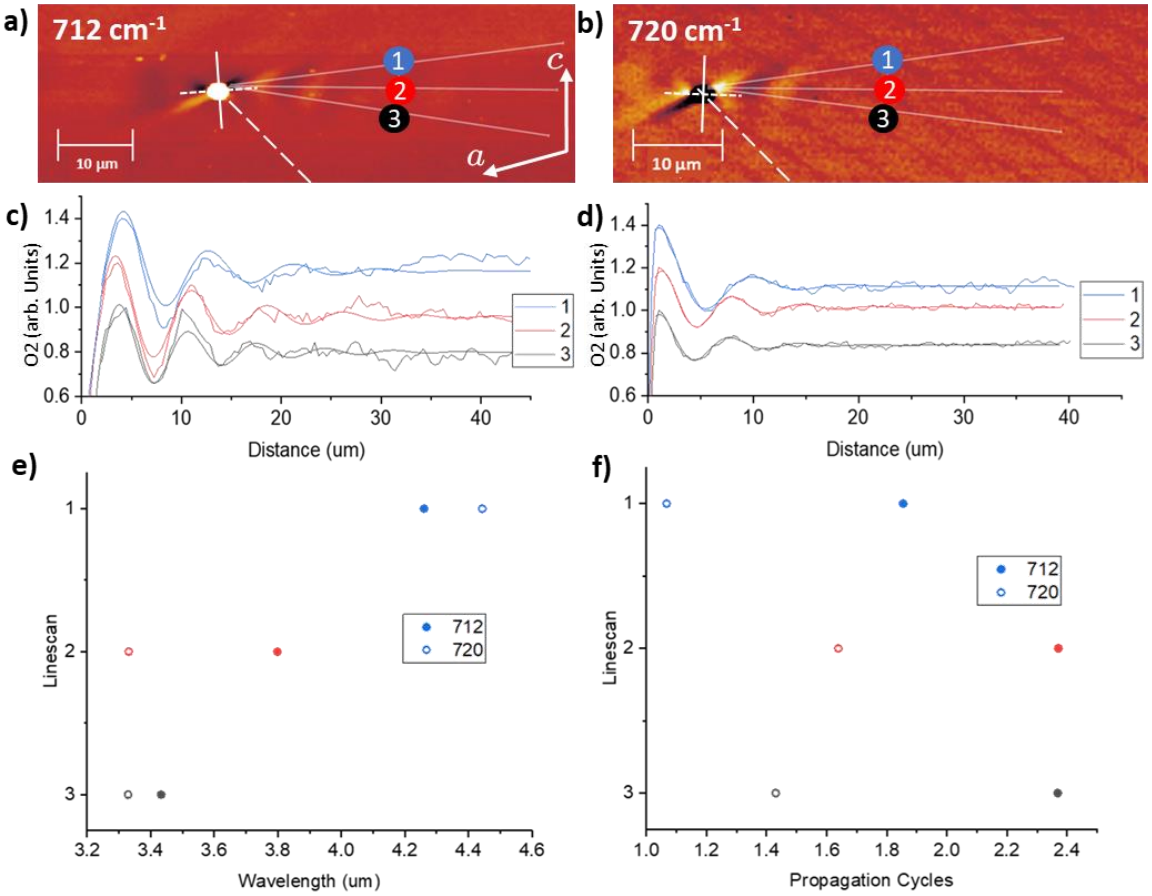

Supplementary Figure 3 – HShP Propagation Length. *a,b) Experimental image of HShPs launched by 2um gold disc at 712cm^{-1} (712cm^{-1}), with 3 linescans taken from the disc along the HShP propgagation fringes. c-d) Linescans from a-b, fitted with a decaying sine wave. e) Polariton wavelength from fitted linescans in a-d, showing wavelengths ~3-4 µm, with the wavelength being longest along the top edge of the hyperbola due to the shear tilt. f) Number of propagation cycles (propagation length divided by wavelength (e)) from fitted linescans (a-d).*

Supplementary Note 5: Frequency varying measurements of bGO polaritons launched from different antenna sizes

In Figure 4 of the main text, we demonstrate the frequency-dependent rotation of the shear polaritons launched by a 2 µm gold disc antenna. Here, we show similar measurements on 1 µm (Supplementary Fig. 4) and 4 µm gold discs (Supplementary Fig. 5).

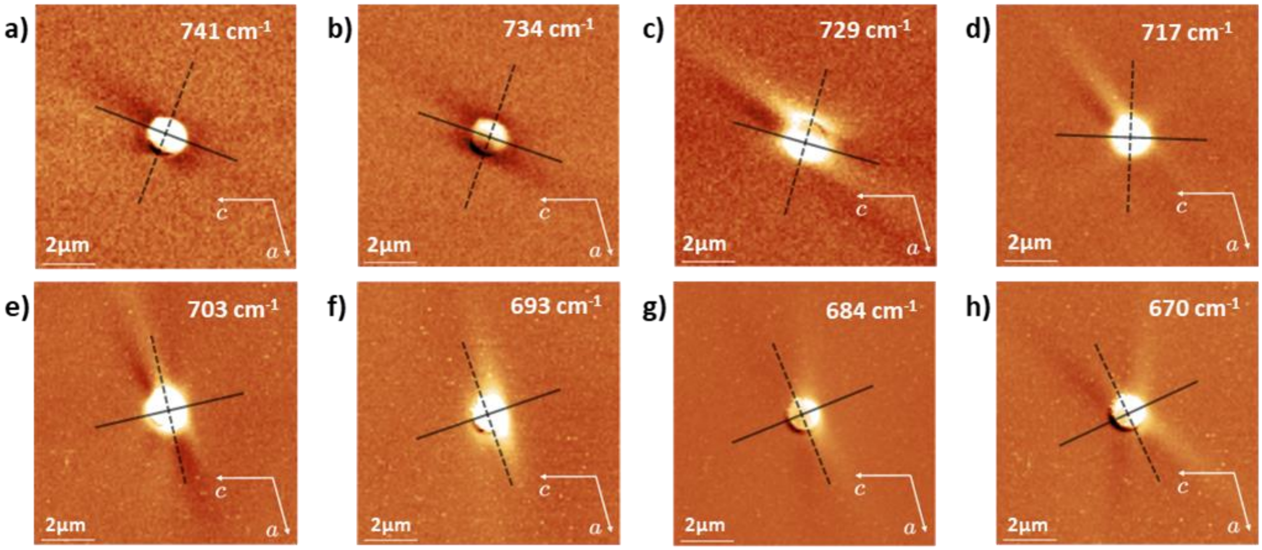

Supplementary Figure 4 – Frequency dependent rotation of HShP propagation from 1-µm discs. *a-h) Rotated polariton axis plotted, as in Figure 4 of the main text, against s-SNOM collected near-field plots of HShPs launched by a 1-µm diameter gold disc, at varying frequencies (Scale bar = 2 µm).*

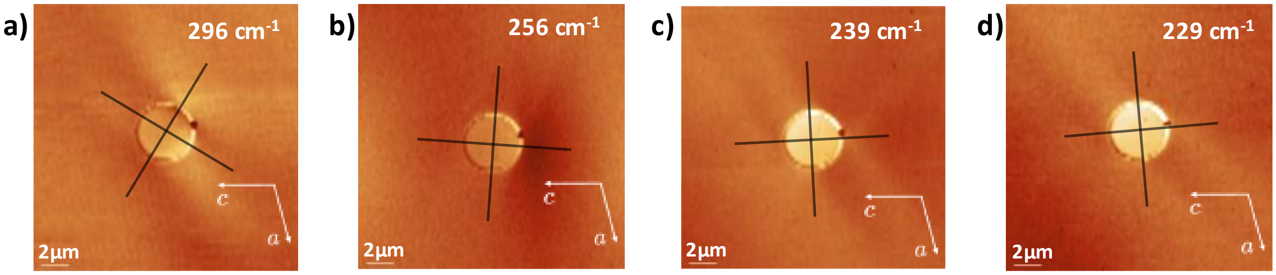

Supplementary Figure 5 – Frequency dependent rotation of HShP propagation from 4-µm discs. *a-d) Rotated polariton axis plotted, as in Figure 4 of the main text, against s-SNOM collected near-field plots of HShPs launched by a 4-µm diameter gold disc, at varying frequencies (Scale bar = 2 µm).*

Supplementary Note 6: Finite element modeling concerning the two mechanisms

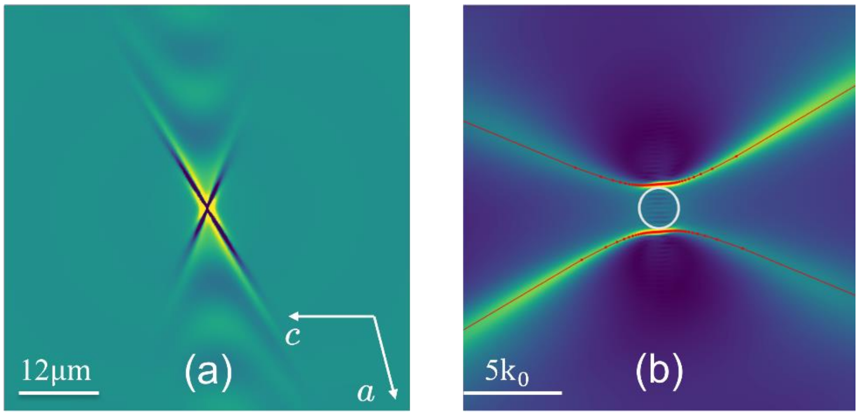

Supplementary Figure 6 – Dipole excitation of HShPs. *(a) Finite element modeling of HShPs propagation excited by dipole source at frequency of 712* cm^{-1}*. (b) Fourier transform of image in (a). The red curves in (b) are the dispersion of HShPs calculated from theory, and the white circle represents the free space light cone.*

As we explained in the main text, the asymmetry propagation of HShPs in disc-launcher excitation arises from two mechanisms: the first mechanism comes from the off-diagonal imaginary component of permittivity tensor, causing the inherent asymmetry in polariton propagation, as seen in near-field point-dipole excitations in Supplementary Fig. 6. The second mechanism is the orientation-dependent phase matching condition between the illuminated disc launcher and the excited polaritons. For example, when alignment of the incident light direction to the one of the asymptotic lines of the hyperbolic wave in real space, the hyperbolic waves in the other branch of asymptotic line get stronger excitation. To verify asymmetry effect from the second mechanism, we perform finite element simulations for disc-launcher excitation with and without the intrinsic shear mechanism. To control and remove the intrinsic shear effect in the simulation, we first diagonalize the real permittivity tensor of bGO by the following procedure

$$\begin{pmatrix} \epsilon'_{xx} & \epsilon'_{xy} & 0 \\ \epsilon'_{xy} & \epsilon'_{yy} & 0 \\ 0 & 0 & \epsilon_{zz} \end{pmatrix} = \mathrm{R}(\omega) \begin{pmatrix} \epsilon_{xx} & \epsilon_{xy} & 0 \\ \epsilon_{xy} & \epsilon_{yy} & 0 \\ 0 & 0 & \epsilon_{zz} \end{pmatrix} \mathrm{R}^{-1}(\omega), \qquad (16)$$

where $\mathrm{R}(\omega)$ is the SO(3) matrix and its rotational angle depends on the frequency ω. As a result, ϵ'_{xy} is an imaginary number. Next, we manually insert a tuning parameter f in front of ϵ'_{xy}, and rotate matrix in the left side of Eq. (16) back to the original coordinate frame

$$\begin{pmatrix} \epsilon''_{xx} & \epsilon''_{xy} & 0 \\ \epsilon''_{xy} & \epsilon''_{yy} & 0 \\ 0 & 0 & \epsilon_{zz} \end{pmatrix} = \mathrm{R}^{-1}(\omega) \begin{pmatrix} \epsilon'_{xx} & f * \epsilon'_{xy} & 0 \\ f * \epsilon'_{xy} & \epsilon'_{yy} & 0 \\ 0 & 0 & \epsilon_{zz} \end{pmatrix} \mathrm{R}(\omega). \qquad (17)$$

Consequently, when $f = 1$, the permittivity tensor in the left side of Eq. (17) becomes the original permittivity tensor. When $f = 0$, it preserves the optical response of the original tensor but gets rid of the

intrinsic shear effect. We deploy the permittivity tensor in the left side of Eq. (17) in COMSOL simulation with and without intrinsic shear effects by tuning f between 1 and 0. As a result, the asymmetry propagation in the case $f = 0$ (Supplementary Figure 7a,b) is solely caused by the orientation-dependent phase matching between the illuminated disc and polaritons wave. When we turn on the intrinsic shear effect, the asymmetry effect of HShPs is enhanced (Supplementary Figure 7c,d) because the two mechanisms contribute to the shear-asymmetry effect constructively.

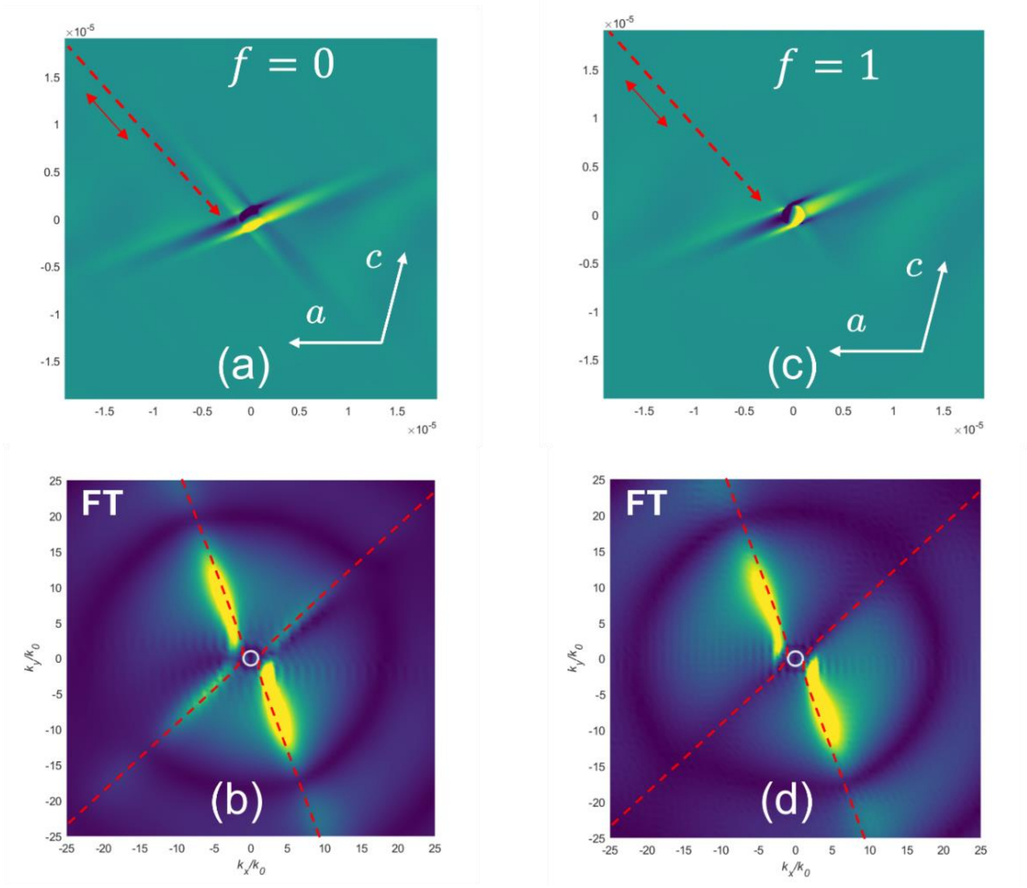

Supplementary Figure 7 – Influence of shear on HShP. *Compare the disc-launcher excitations with and without intrinsic shear effect. (a,c) Finite element modeling of HShPs propagation by disc-launcher excitation at frequency of 720* cm^{-1} *without (a) and (c) with intrinsic shear effect. (b,d) Fourier transform of image in (a,c). The red dashed arrow represents the orientation of illuminating light.*

Supplementary Note 7: FOM Calculation

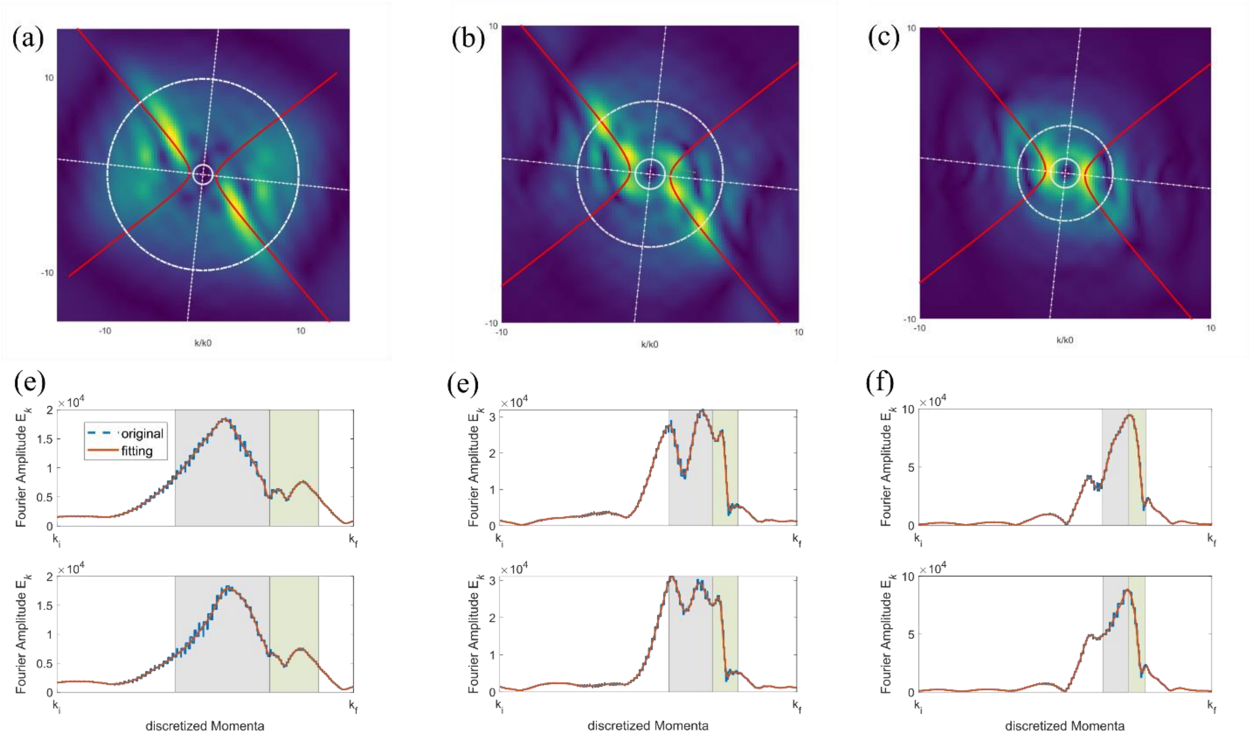

Supplementary Figure 8 – Analyzing FOM of Experimental HShPs. *Sampling the experimental Fourier spectrum of HShPs excitation by illuminating gold disc-launchers of different size. (a-c) Fourier spectra of experimental near-field images of HShP propagation launched by a (6,4,2) μm gold disc at a frequency of 720* cm^{-1}*, with illumination incident from the lower left corner. (e-f) 1D Fourier intensity distribution extracting along the IFCs of HShPs for various disc-launchers in (a-c). White circles in (a-c) intersecting dispersion of HShP determine the upper bound of the integration in k-space of FOM calculation, and the radius of the circle is calculated by Eq. (13). Light green (grey) shaded regions in (e-f) correspond to the odd (even) quadrants in k-space in Supplementary Fig. 1.*

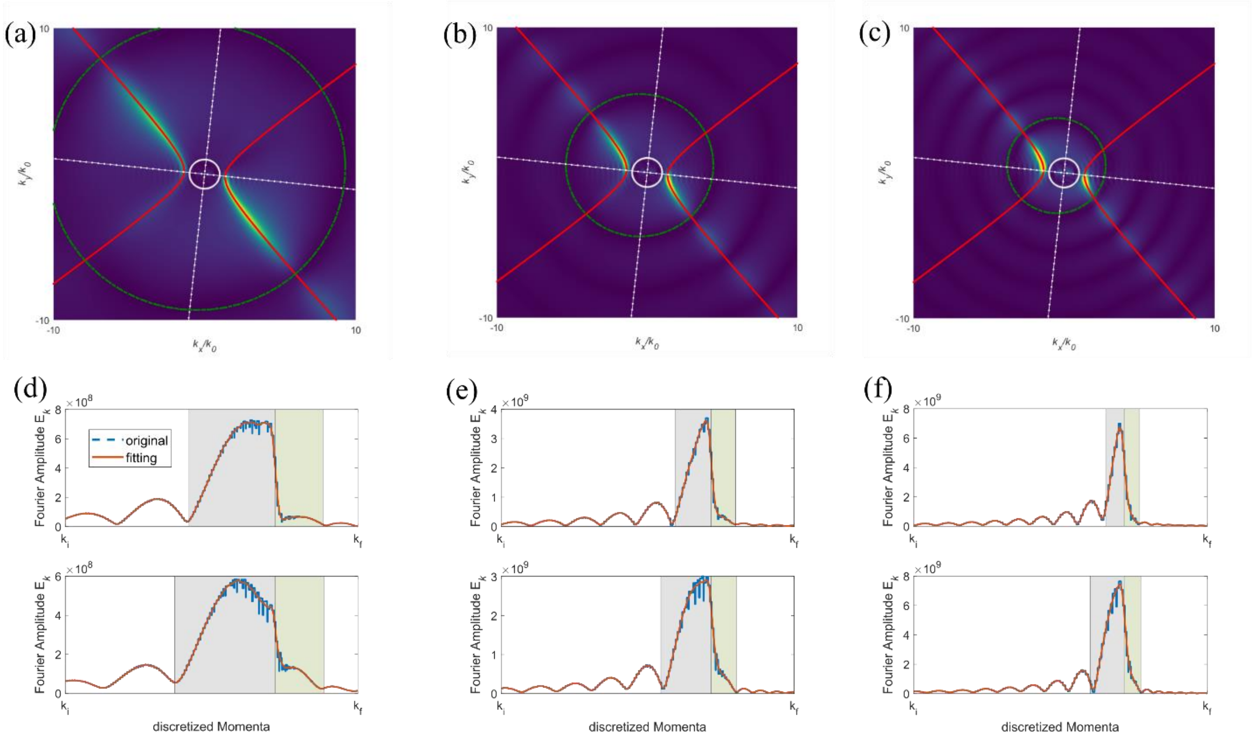

Supplementary Figure 9– Analyzing FOM of Simulated HShPs. *Sampling the simulated Fourier spectrum of HShPs excitation by illuminating gold disc-launchers of different size.*

Supplementary Note 8: Simulating shear polariton propagation at various frequencies

We also calculated the polariton propagation from a 2 µm gold disc for select frequencies from the main text Figure 4 (Supplementary Fig. 10). These show excellent agreement at varying excitation frequencies. In particular, the Fourier-space profile (Supplementary Fig. 10i-l) illustrates the frequency tuning of the HShP hyperbola, and the change in the degree of asymmetry (stemming from both the dispersion and the orientation of the hyperbolic axis with respect to the incident beam).

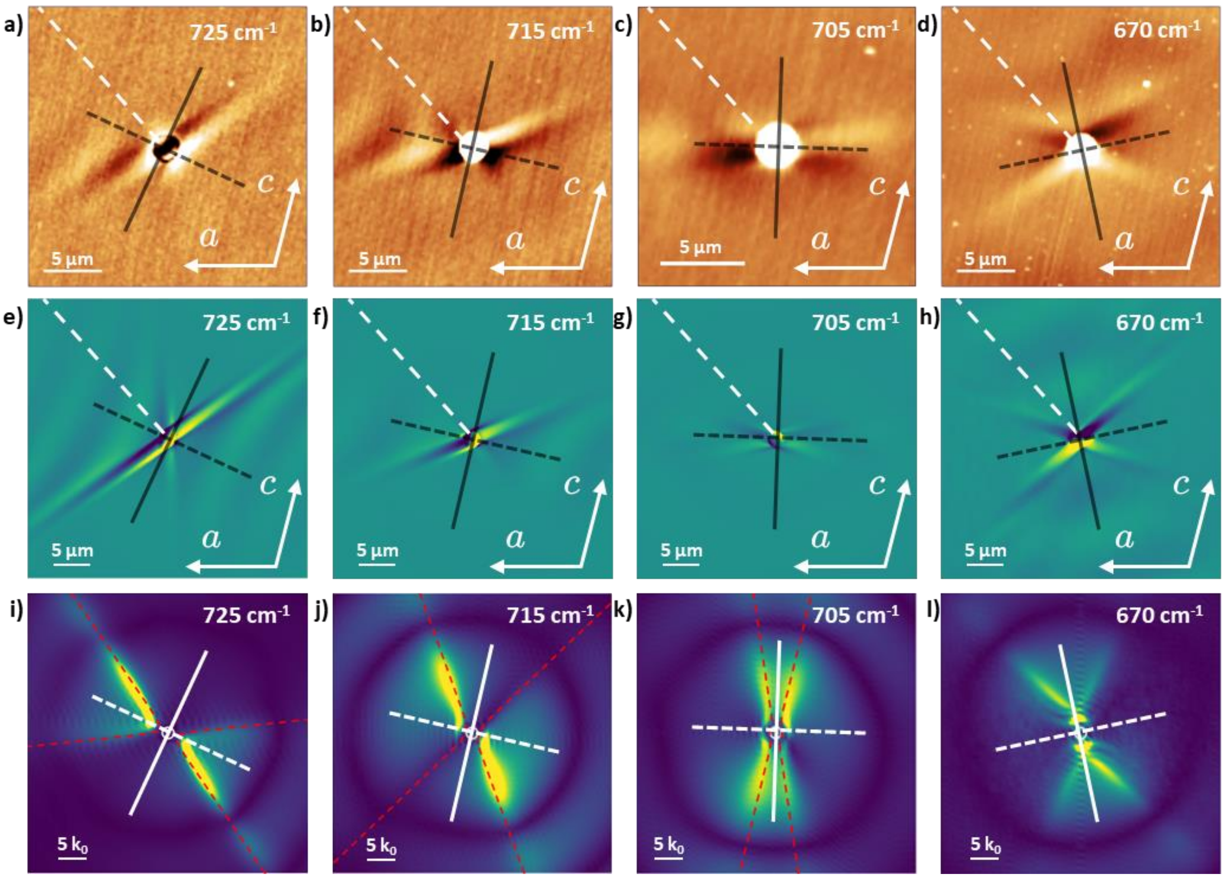

Supplementary Figure 10 – Comparison of experiment to simulated HShPs at varying frequency. *a-d) Select frequencies from main figure 4 (rotated so that the a-axis is horizontal) . e-h) corresponding COMSOL simulations of the HShP propagation. i-l) Fourier transform of the electromagnetic HShP simulations in (e-h). Plots are overlaid with crossbars indicating the axis of the HShP hyperbola.*
